# Electronic cigarette for smoking cessation: a *fast-track *Delphi consensus of French-speaking experts

**DOI:** 10.1186/s13690-025-01725-x

**Published:** 2025-10-23

**Authors:** Emmanuelle Lüthi, Camille Velarde Crézé, Luc Lebon, Isabelle Jacot-Sadowski, Karin Zürcher, Jacques Cornuz, Olivier Duperrex

**Affiliations:** 1 Departement of Ambulatory Care, University Center for Primary Care and Public Health (Unisanté), University of Lausanne, Route de Berne 113, Lausanne, 1010 Switzerland; 2 Department of Health Promotion and Prevention, University Center for Primary Care and Public Health (Unisanté), University of Lausanne, Lausanne, Switzerland

**Keywords:** Electronic nicotine delivery system, Vaping, Smoking cessation, Nicotine, Delphi technique, *Fast-track* Delphi, Consensus

## Abstract

**Background:**

The French-speaking Society for Smoking Cessation (*Société francophone de tabacologie*, SFT), which promotes scientific knowledge and training on smoking cessation, asked us to identify and quantify consensual agreements among its members on the use of e-cigarette for smoking cessation.

**Methods:**

We used the *fast-track* Delphi process, starting with a modified version of the Nominal Group Technique where experts generated propositions to answer the target question: “What is the place and usefulness of vaping in the clinical care of people who smoke?”, followed by two Delphi surveys (e-questionnaires), in which they expressed their opinion on each statement (9-point Likert-scale) and commented. Consensual agreement is reached on a statement with a median ≥ 7 on the 1–9 Likert-scale (agreement) and an interquartile range (IQR) ≤ 3 scale points (consensus).

**Results:**

Over 40 days in autumn 2023, 87/163 (53%) experts reached consensual agreement on 26/33 (79%) statements, notably on the main outcome “According to current data, the e-cigarette is effective for smoking cessation (abstinence of 6 months or more)” (median 7, IQR 6.5–7.5). They considered that e-cigarette very likely reduces the risks compared to smoking (8, 7–9), and "can be used in association with nicotine replacement products" (9, 8–9). No consensual agreement was reached for adverse effects and potential benefits for subgroups (i.e. pregnancy, hospitalized).

**Conclusions:**

Participating experts agreed that e-cigarette can help to quit smoking. Agreeing on priority actions for smoking cessation is a base to develop clinical guidelines and trainings, and to tackle the industry-led tobacco epidemic.

**Supplementary Information:**

The online version contains supplementary material available at 10.1186/s13690-025-01725-x.

**Table:** 

Text box 1. Contributions to the literature
• This study contributes to the debate on the use of e-cigarette by showing that French-speaking experts agreed that it is effective for smoking cessation and can contribute to harm reduction.
• Findings of this *fast-track* Delphi process can guide healthcare professionals to support smoking cessation and can be used to develop clinical guidelines and trainings.
• Such structured consensus processes can contribute to avoid industry-led divisions among the public health community about tobacco and nicotine products, to inform policymakers, and to support informed and balanced decisions.

## Background

Electronic cigarette (e-cigarette or electronic nicotine delivery systems, ENDS) enables the delivery of nicotine through the process of vaping – when the user inhales or presses a button, the battery activates the heating element, which vaporizes the e-liquid, creating an aerosol that the user inhales – a process which permits consuming nicotine without any use of tobacco. Several generations of e-cigarette have consecutively appeared on the market in the last two decades. Due to the absence of tobacco combustion and associated harmful effects, e-cigarette expose users to much lower levels of toxic chemicals [1, 2]. For this reason, e-cigarette can be viewed as a harm reduction option [3]. However, the evidence of using e-cigarette is still limited to short term health effects, the long-term ones remaining poorly documented [4, 5]. Clinical trials evaluating e-cigarette for smoking cessation showed throat and mouth irritation, headache, cough, and nausea as the most commonly reported adverse events. Based on a small number of studies and a follow-up of two years maximum, no evidence of serious harm from e-cigarette with nicotine was reported [6, 7].

Despite the growing evidence [6–8] of the potential of e-cigarette to help people stop smoking, the use of e-cigarette in the clinical care of people who smoke is still a matter of debate among tobacco control specialists, at national and international levels [6–10]. Yet, the population, policymakers and many patients are asking healthcare professionals about the added value of vaping in the process of smoking cessation. This, in turn, triggers a need for healthcare professionals to have recommendations on the use of e-cigarette for smoking cessation.

To define its institutional position on the use of e-cigarette for smoking cessation, and therefore support clinical activity, the French-speaking Society for Smoking Cessation (*Société francophone de tabacologie*, SFT) needed to understand first on which aspects its members agreed or not. It gave us the mandate to help identify and quantify consensual agreements amongst their members on the use of e-cigarette for smoking cessation.

When scientific evidence is lacking for health recommendations, methodologies like Delphi process to build a consensus are useful [11, 12]. Often used to obtain consensus when participants cannot meet easily, the Delphi technique allows participants to take position on a topic, through iterative questionnaires with open-ended and closed questions. Answers from each round are used to build the next questionnaire, refining the elements on which a consensus is reached or not [13, 14]. These consultations often take months to be completed, which can be viewed as a limitation.

In 2022, we developed and tested a *fast-track* Delphi process, combining nominal group technique (NGT, step 1) and conventional Delphi aspects (steps 2 and 3), to identify and quantify areas of consensus and dissensus among experts in a very short time [15, 16]. This hybrid process is a useful compromise between methodological rigor, rapidity constraints and context coherence. We used this new methodology to develop consensual agreements among members of the SFT at its request.

## Methods

The study protocol was not prospectively registered, and there was no pilot study. All communication, sessions and questionnaires were in French.

### Research team

The research team consisted of experts in smoking cessation and tobacco control (LL, IJS, KZ, JC) and experts in the *fast-track* Delphi process (CVC, EL, OD) [15, 16]. Most of the team have experience in using NGT and Delphi techniques to obtain consensus. The team pilot tested the technical aspects of the e-questionnaire but none of the team responded to the final versions.

### Participants

The authors considered all members of the SFT to be experts in smoking cessation. According to McPherson and colleagues (2018), an expert is a person who has special skills and/or knowledge derived from training or experience in a specific field [17]. SFT members are from France, Belgium, Luxembourg, Switzerland, Canada, Burkina Faso and Mali. Patients, carers or members of the public were not involved in the design or conduct of the study. As participants were experts, no ethical approval was needed. Consent from participants was tacit. Participants provided their email addresses for the e-questionnaires to allow production and emailing of individualised reports. We guaranteed confidentiality by limiting access to participants identity to the three persons in the team involved in the production and distribution of the individualised reports. Participants received no compensation. Research team did not contribute to voting.

### Preparatory research and target question

As participants were experts in the field, we did not conduct a preliminary literature review. Based on the mandate, the authors defined the target question to be addressed throughout the whole process: “What is the place and usefulness of vaping in the clinical care of people who smoke?”.

### *Fast-track* Delphi process

We used the *fast-track* Delphi process [15, 16] which has three steps:

- Step 1: a group session by videoconference using an adapted NGT to generate thematic proposals and prioritize them
- Step 2: e-questionnaire for experts to quantify their opinion and comment (see Additional file 1)
- Step 3: e-questionnaire for experts to re-quantify their opinion in light of group results (see Additional file 1)

### Consensus definition

We used a Likert-scale from 1 (total disagreement) to 9 (total agreement) to quantify the level of agreement. For each statement, we considered the *consensual agreement reached* if both the median was 7 or more on the 1–9 Likert-scale (agreement) and the interquartile range (IQR) 3 scale points or less (consensus). The threshold for disagreement was a median of 3 or less. We considered as definitive the statements that had reached the thresholds of consensual (dis)agreement, and for which most experts comments did not indicate the need for reformulation [15].

### Data collection and analysis

#### Step 1 – Participants and Nominal group technique (NGT)

For step 1, the SFT Board of Directors and Executive Committee received the target question by email together with the invitation to a videoconference meeting.

The NGT consists of four phases and is used to structure a group brainstorming meeting [18, 19]. Here we used a modified version of this technique to: (a) generate thematic proposals in response to the target question detailed above; then (b) select some of these thematic proposals according to their degree of priority. The following phases were carried out during a group meeting lasting two hours and led by three people (an expert in the *fast-track* Delphi process and two experts in smoking cessation and tobacco control):

- Individual idea generation (phase 1): experts worked individually to put down on paper all the ideas that came to mind in response to the target question
- Pooling (phase 2): several successive rounds of discussion were held, during which the experts took it in turns to state one of their ideas generated in phase 1. Table rounds continued until there were no longer new ideas
- Discussion (phase 3): under the moderation of the session moderators, experts proposed a clarification of their ideas listed in phase 2; ideas then became thematic proposals. Whenever relevant, those proposals were also grouped into categories. This was therefore a discussion about structuring and clarifying the proposals, not a debate of opinion
- Prioritization vote (phase 4): each expert anonymously selected the 20 thematic proposals that he/she felt should be considered during step 2 of the *fast-track* Delphi process, using the online application Wooclap. Proposals with 3 votes or less were excluded

Throughout the meeting, we used the MindManager software (version 22.2.366) to organize propositions and statements and projected them continuously so that the experts could see the gradual construction, classification, and clarification of the thematic proposals.

#### Steps 2 and 3 – Participants, e-questionnaires and analysis

For step 2 e-questionnaire, all SFT members who had paid the annual membership as of 5 October 2023 (including those involved in the step 1) received by email the link for the e-questionnaire and were asked to complete it within eleven days (with a reminder sent to all after 6 days).

For step 3 e-questionnaire, only responders to step 2 questionnaire received the link and were asked to complete it within six days (with a reminder sent to non-responders after 3 days). Due to holidays and the initially rather low response rate, the deadline to complete the e-questionnaire of step 3 was extended by seven days.

The definitions of e-cigarette (considered only with nicotine) were indicated at the beginning of both e-questionnaires for the purposes of the survey. Both e-questionnaires were accessible on an institutional REDCap [20, 21].

To develop the step 2 e-questionnaire, we slightly rephrased the thematic proposals generated and selected during step 1 (NGT) and organized them into four thematic sections (scientific knowledge, advice for clinical care, specific populations, miscellaneous).

Each selected proposal was included in the e-questionnaire as an affirmative statement, and experts were asked to quantify their opinion on each statement on a Likert-scale from 1 (total disagreement) to 9 (total agreement) and could add a free-format text comment to each statement. A section of the step 2 e-questionnaire also addressed the profile of the experts (gender, age, professional field activity, clinical activity location, medical consultation type, training, country of practice).

The step 3 e-questionnaire was developed according to responses and comments from the previous one. All statements not considered as definitive were rephrased and included in the e-questionnaire of step 3, allowing the experts to re-quantify and comment each statement. Experts were instructed to consult their individualized report showing their responses to the statements in step 2 (see Additional file 2) so they could consider the group results and comments from the previous step, contributing to the development of consensual agreements.

We used the previously developed R code to generate a generic report and one individualized report per expert at the end of each step, and to prepare the final report [15, 16].

## Results

The *fast-track* Delphi process took 40 calendar days in 2023: step 1 on the 27 September, step 2 from 5 to 16 October (11 days) and step 3 from 24 October to 6 November (13 days).

### Step 1—Nominal group technique (NGT)

Eleven of the 23 (48%) experts of the SFT Board of Directors and Executive Committee participated in step 1. They generated 43 statements throughout the phases 1–3 of the NGT process and selected 34 statements in the prioritization vote.

### Steps 2 and 3—E-questionnaires

Then, 163 SFT members with up-to-date membership fee received the link to the step 2 e-questionnaire by e-mail. Eighty-seven members completed it (53% of members). Most respondents were from France, aged 50 to over 60, physicians with clinical activity (mainly ambulatory consultation) in hospitals. Most had the French Inter-University Diploma (DIU) (Table 1). Among these 87 respondents, 48 also completed the step 3 e-questionnaire (55% of step 2). One person answered only to the step 3 e-questionnaire and was thus excluded from the analyses.

**Table 1:** Characteristics of step 2 respondents

	*n* (*N* = 87)	**Proportion**
**Gender**		
Female	53	60.9%
Male	34	39.1%
**Age**		
≥ 60 years old	37	42.5%
50 to 59 years old	22	25.3%
40 to 49 years old	17	19.5%
30 to 39 years old	9	10.3%
20 to 29 years old	1	1.1%
I do not wish to answer	1	1.1%
**Professional activity**		
Physician	61	70.1%
Other health or care professional activity	10	11.5%
Nurse	7	8.0%
Midwife or male midwife	7	8.0%
Pharmacist	2	2.3%
Activities *		
Care	74	85.1%
Prevention	36	41.4%
Teaching	32	36.8%
Research	17	19.5%
Other	5	5.7%
Place of clinical activity *		
Hospital center	56	64.4%
Private practice or general practitioner	20	23.0%
Health or care center	10	11.5%
Other	8	9.2%
Type of consultation *		
Ambulatory consultation	54	62.1%
In-patients	37	42.5%
Training in smoking cessation *		
Inter-University Diploma (DIU)	67	77.0%
Other	28	32.2%
**Country of main professional activity**		
France	80	92.0%
Belgium	5	5.7%
Switzerland	1	1.1%
Other	1	1.1%

Proportions are calculated on the total of respondents

^*^Several answers possible—the sum of the proportions may exceed 100%

**Fig. 1:**
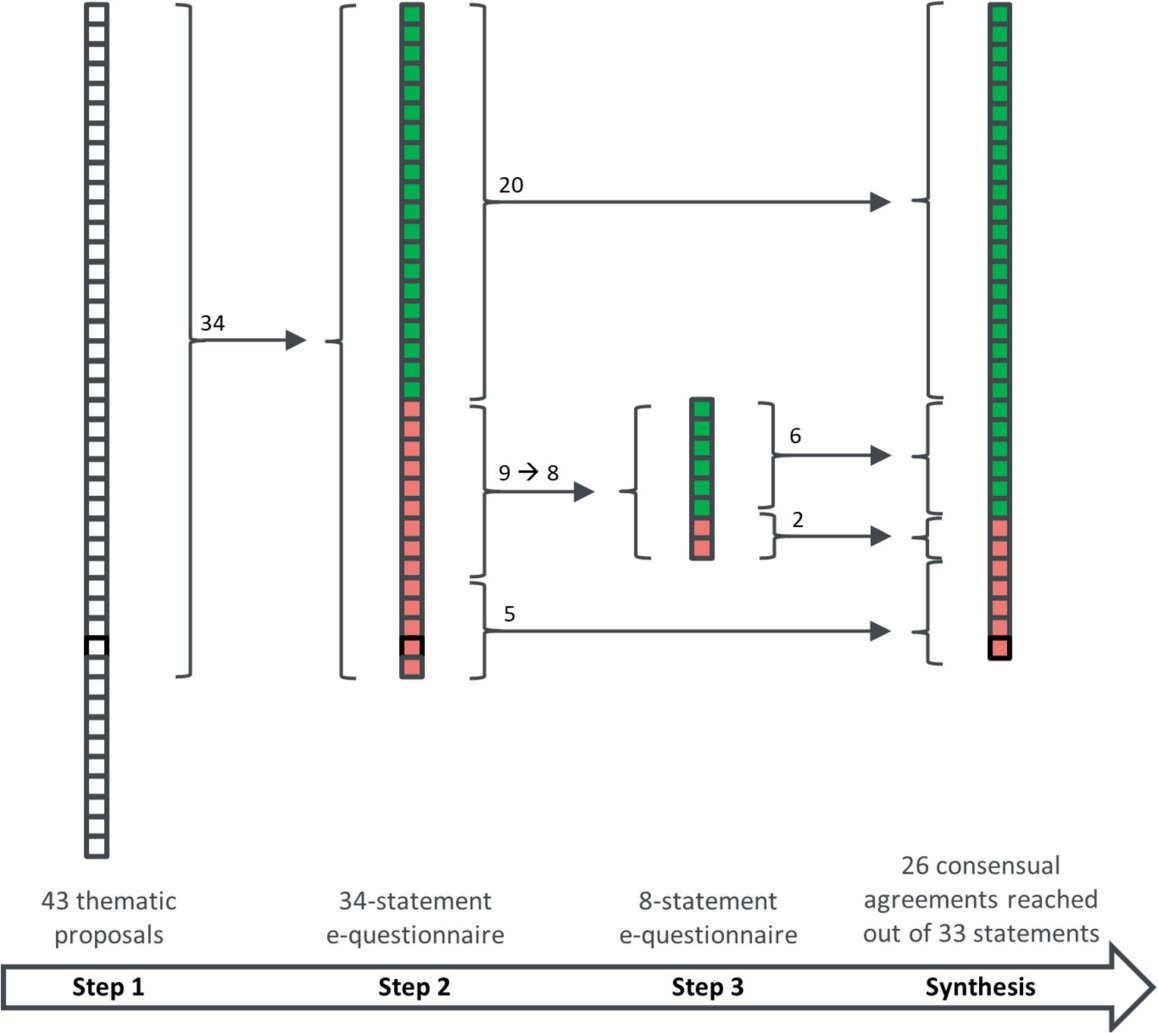
Flow of statements through the three steps of the *fast-track* Delphi process. A square represents a proposal (step 1) or a statement (steps 2 and 3). Green and red squares indicate statements that have reached, or have not reached consensual agreement, respectively

Figure 1 illustrates the flow of thematical proposals and statements throughout the *fast-track* Delphi process.

Upon completion of step 2, 25 out of 34 statements were considered as definitive: 20 (59%) achieved consensual agreement and 5 (15%) did not due to highly divergent comments that precluded unambiguous rephrasing.

The 9 remaining statements did not reach consensual agreement or had comments pleading for a reformulation. Two statements from step 2 were merged into one statement for step 3. At the end of step 3, 6/8 (75%) statements reached consensual agreement.

Over the whole process, 26/33 (79%) statements reached consensual agreement between experts.

Tables 2 and 3 show the statements having reached or not reached consensual agreement, respectively, together with their statistical data (median and IQR) and a graphical distribution of experts’ responses on the 1 to 9 Likert-scale.

**Table 2:** Statements having reached a consensual agreement at the end of step 2 or 3 e-questionnaire

Consensual agreement reached	Median	[IQR](min-max)	(1) total disagreement. total agreement (9)
**Scientific knowledge**			
The action of the e-cigarette on smoking cessation is well known	7	[6-8](1-9)	
The benefit-risk ratio of e-cigarette use in the context of smoking cessation aid is favorable	7	[7-9](1-9)	
According to current data, the e-cigarette is effective for smoking cessation (abstinence of 6 months or more)^a^	7	[6.5-7.5](2-9)	
The e-cigarette is very likely to reduce the risks (morbidity and mortality) of smoking, provided you stop smoking altogether^a^	8	[7-9](3-9)	
**Advice on clinical care**			
The e-cigarette is an important tool in the clinical care of people who smoke	7	[5-8](1-9)	
The e-cigarette can be used as nicotine replacement therapy	7	[6-8.25](1-9)	
The e-cigarette has a place and a usefulness in smoking cessation, provided it is used under the right conditions (nicotine dose, type of liquid, electrical resistance, etc.)	8	[7-9](1-9)	
The e-cigarette can be used in conjunction with nicotine replacement products	9	[8-9](1-9)	
The e-cigarette should be used for a limited period, with the aim of quitting in a second phase, once smoking cessation has been consolidated	8	[7-9](3-9)	
The simultaneous use of conventional cigarettes and the e-cigarette (dual use) should only be a transitional phase, with the aim of stopping smoking altogether	9	[7-9](1-9)	
Smoking cessation specialists must be trained to provide information as well as medical and technical advice on the use of the e-cigarette	9	[7.5-9](4-9)	
Patients should be encouraged to try different flavors	7	[5-8](1-9)	
It is necessary to monitor the undesirable effects of the e-cigarette	9	[8-9](2-9)	
Pharmacological treatments and the e-cigarette need to be presented to people who smoke, with clear explanations of their advantages and disadvantages, and then the person who smokes needs to be supported in their choice (shared decision making)^a^	8	[7-9](1-9)	
The e-cigarette is an option for helping people to stop smoking, when they want to vape or refuse pharmacological treatments^a^	8	[7-9](4-9)	
The e-cigarette can be recommended in situations where there is a risk of relapse, such as when going out in the presence of other people who smoke^a^	8	[7-9](2-9)	
In the event of withdrawal symptoms when using an e-cigarette, it is recommended to increase the nicotine concentration, not its electrical power^a^	8	[7-9](1-9)	
**Specific populations**			
The e-cigarette can be recommended for smoking cessation in patients with psychiatric disorders	8	[7-9](1-9)	
The e-cigarette can be recommended for smoking cessation in patients with co-addictions	8	[7-9](1-9)	
A pregnant woman who has quitted smoking with an e-cigarette should not be discouraged from using it if there is a risk of relapse	8	[7-9](1-9)	
A breast-feeding woman who has quitted smoking with an e-cigarette should not be discouraged from using it if there is a risk of relapse	8	[6-9](1-9)	
The e-cigarette can be recommended for smoking cessation in hospitalized patients	7	[5-8](1-9)	
The e-cigarette can be recommended for smoking cessation in coronary patients	7	[5-8](1-9)	
The e-cigarette can be recommended for smoking cessation in COPD patients	7	[5.25-8](1-9)	
**Miscellaneous**			
There is a need for clinical guidelines on the use of the e-cigarette for smoking cessation	9	[7-9](1-9)	
Clinical recommendations should distinguish between products from the tobacco industry and those from independent manufacturers	9	[7-9](1-9)	

^a ^Statements from the step 3 e-questionnaire

**Table 3:** Statements not having reached a consensual agreement at the end of steps 2 or 3 e-questionnaires

Consensual agreement not reached	Median	[IQR](min-max)	(1) total disagreement. total agreement (9)
**Scientific knowledge**			
According to current data, the e-cigarette use over several years produces few severe adverse effects^a^	6	[3-7](1-9)	
Nicotine dependence is comparable between people who smoke and recent e-cigarette users^a^	6	[3-7](1-9)	
**Specific populations**			
The e-cigarette can be recommended for peri-operative smoking cessation (pre-and post-operative)	7	[5.75-9](1-9)	
The e-cigarette can be recommended for smoking cessation pregnant women	5	[2-7](1-9)	
The e-cigarette can be recommended for smoking cessation in breast-feeding women	6	[2.25-7.75](1-9)	
The e-cigarette can be recommended for smoking cessation in patients under 18	4	[2-7](1-9)	
**Miscellaneous**			
The e-cigarette should only be sold in pharmacies	5	[1-7](1-9)	

^a ^Statements from step 3 e-questionnaire.

### Scientific knowledge

Regarding scientific knowledge, the SFT experts reached consensual agreement on the fact that e-cigarette is effective in smoking cessation (median 7 [IQR 6.5–7.5]), its benefit-risk ratio is favourable (7 [7–9]) and it is also very likely to reduce the risks of smoking (if tobacco consumption is stopped) (8 [7–9]).

They did not reach consensual agreement concerning severe long-term adverse effects (6 [3–7]) and nicotine dependence (6 [3–7]).

### Advice on clinical care

In clinical care of people who smoke, the SFT experts considered that e-cigarette is an important tool (7 [5–8]), can be used as nicotine replacement therapy (7 [6–8.25]) and in association with nicotine replacement products (9 [8, 9]), provided it is used under the right conditions (nicotine dose, type of liquid, electrical resistance, etc.) (8 [7–9]), but that it should be used for a limited period, with the aim of stopping once smoking cessation has been consolidated (8 [7–9]).

Experts agreed that people who smoke should be offered both pharmacological treatments and e-cigarette, with clear explanations of the advantages and disadvantages (8 [7–9]). To do so, specialists must be trained to provide information as well as medical and technical advice on the use of e-cigarette (9 [7.5–9]). For instance, e-cigarette can be recommended in situations where there is a risk of relapse, such as when going out in the presence of other people who smoke (8 [7–9]).

In the event of withdrawal symptoms when using e-cigarette, there was consensus to increase the nicotine concentration, not its electrical power (8 [7–9]). The simultaneous use of conventional cigarettes and e-cigarette (dual use) should only be a transitional phase, with the aim of stopping smoking altogether (9 [7–9]). All statements in this thematic section reached consensual agreement.

### Specific populations

Regarding specific populations, e-cigarette may be recommended for smoking cessation in patients with psychiatric disorders and co-addictions (8 [7–9]), with chronic obstructive pulmonary disease (COPD) (7 [5.25–8]), coronary heart disease as well as hospitalized patients (7 [5–8]).

There was no consensual agreement on recommending e-cigarette for smoking cessation to minors under 18 (4 [2–7]), to pregnant (5 [2–7]) or breastfeeding women (6 [2.25–7.75]) and to pre-and post-operative patients (7 [5.75–9]). However, a pregnant (8 [7–9]) or breastfeeding woman (8 [6–9]) who has stopped smoking using e-cigarette should not be discouraged from using it if there is a risk of relapse.

### Miscellaneous

Experts concurred there is a need for clinical recommendations on the use of e-cigarette for smoking cessation (9 [7–9]) and for monitoring of the adverse effects of e-cigarette (9 [8, 9]). They also agreed that clinical recommendations should distinguish between products from the tobacco industry and those from independent manufacturers (9 [7–9]). But there was no agreement that e-cigarette should only be sold in pharmacies (5 [1–7]).

## Discussion

A large majority of statements reached agreement (Table 2).

### Use for smoking cessation

Experts involved in our study considered that e-cigarette can be a useful temporary support to smoking cessation, including some groups (patients with psychiatric disorders, co-addictions, COPD, coronary heart disease or hospitalised). They also agreed that in clinical care e-cigarette is useful as a nicotine replacement tool and in association with nicotine replacement therapies. It is interesting to note that the consensus progressively evolved over time, from e-cigarette not being advised as a first-line treatment to help quit smoking (Delphi study conducted in 2013–2014 in Switzerland [11]; and in 2014 in France [22]), to experts agreeing to see e-cigarette as a mean to quit (Delphi studies conducted in 2016 in France [23]; and in 2017–2018 in the Netherlands [24]), to experts agreeing that health authorities should encourage people who smoke conventional cigarettes to switch to e-cigarette as a harm reduction tool (Delphi study conducted in 2018–2019 in France and Switzerland [12]). Our study confirms this evolution, as experts not only agree that e-cigarette can be used as smoking cessation tool, but also that it can be used in combination with other nicotine replacement therapies.

The present study found stronger and detailed agreement for the use of e-cigarette in smoking cessation compared to the above cited studies. We hypothesise that this is mostly due to the growing evidence from scientific studies. For instance, experts cited the Cochrane reviews from 2022 and 2023 about 20 times in their comments [7, 25]. Incidentally, an update was published after our data collection [6, 8], as well as two new randomised clinical trials [26, 27].

### Knowledge and training

The experts involved in this study confirmed there is a need for monitoring the adverse effects of e-cigarette. This is in line with a previous consensus of international experts [12], despite other previous consensus that e-cigarette is not dangerous to the health of people who smoke tobacco [11] or is a harm reduction tool compared to conventional cigarettes [10, 12].

Consensus was also reached about the need for clinical recommendations on the use of e-cigarette for smoking cessation. This is in line with a recent Australian study and two reviews finding that clinicians need to be accompanied by a training effort to provide information as well as medical and technical advice on the use of e-cigarette [28–30].

### Minors and policies

There was no consensual agreement on recommending e-cigarette for smoking cessation to minors (under 18), in line with a previous consensus that disposable electronic cigarettes are a public health issue for children [15].

There was no consensual agreement on recommending e-cigarette for smoking cessation to pregnant or breastfeeding women. However, experts agreed that a pregnant or breastfeeding woman who has quit smoking using e-cigarette should not be discouraged from using it if there is a risk of relapse. By contrast, a 2021 consensus in Canada stated that pregnant or breastfeeding women who quit smoking and have continued vaping, should be advised to quit vaping [31].

There was no agreement that e-cigarette should only be sold in pharmacies. Our experts mentioned a certain number of advantages of restricting the selling of e-cigarette to pharmacies only, acknowledging the fact that this might be possible only if e-cigarette is registered as a drug: this could a) enable a better monitoring of people who want to stop smoking and provide them with the correct dosage and use information, provided that pharmacy staff maintain an appropriate knowledge and skills, b) limit the power of the tobacco industry, and c) limit access for minors to prevent nicotine use. On the other hand, experts also mentioned several disadvantages, the main one being that there is a need for maintaining a high level of accessibility for people who want to stop smoking using e-cigarette, especially for those who do not want to medicalise their cessation attempt. In 2024, Australia implemented a policy reform permitting the distribution of e-cigarettes by pharmacists to individuals aged 18 years and over without the need for a medical prescription. [32]. The monitoring of its impact might provide insights for future policies.

### Strengths, limitations and perspectives

The e-cigarette can lead to lively debate, particularly as it has the potential to help people stop smoking on the one hand; and to encourage people who do not smoke, especially young people, to take up smoking on the other hand. The *fast-track* Delphi process allows to overcome some of these debates, by allowing all experts to express their opinion and bring out points of agreement (and disagreement), in a fairly short time frame (40 days instead of several months with conventional Delphi study) on subjects where knowledge is evolving rapidly, such as e-cigarette. Furthermore, in the *fast-track* Delphi process, two data components are considered: the agreement and the dispersion of the responses (consensus), which ensures the robustness of the method. Another strength of our study compared to conventional Delphi study is the individualized report after step 2, which allows the participants to situate themselves on the visual scale and to read all the others participants’ comments, as well as the step 1 elaborating and clarifying statements. Finally, the use of technical support such as R code allows the acceleration of the process. There are several limitations to our study. Given our recruitment method, this constitutes convenience sampling. We could only access part of professional profiles of members (i.e. membership subscription information), which enabled us to make useful yet limited comparisons between participants and the whole SFT members [see Additional file 3]. Both profile distributions are similar except for the proportion of “other health or care professional activity”, which seems slightly overrepresented in the participant sample, and the gender proportions. For the latter, we have yet no reason to assume that it could have biased participant responses distribution. As explained in the method, ‘consensual agreement’ refers to statements with high agreement and little dispersion. It is a useful measure of strong agreement, but it does not mean unanimity [16].

Our results represent a consensual agreement in a specific field (members of the SFT), time and context. Indeed, when new studies are published, policies defined or products marketed, experts may change their opinions. Moreover, the response reflects some French-speaking countries (France, Belgium, Switzerland, Tunisia) and might not be applied to others. Finally, our results constitute a consensual agreement among respondents active in health care and may not represent opinion of lay people. We acknowledge the importance to invite lay people to reach a more meaningful and relevant research in a future *fast-track* Delphi process [33]. In that case, the methods would be applied as such, but the target-question(s) would be adapted to the profile of the target participant group. The views of lay people are usually gathered through opinion polls or official referendums. A Delphi would allow a more participative definition of the answers.

New tobacco and nicotine products spread debate and divisions among the public health community [34]. Therefore, producing independent research, finding agreements and focusing on priority actions is crucial to tackle the industry-lead tobacco epidemic [35].

Further research is needed to assess the adverse and long-term effects of e-cigarette, as well as to create clinical recommendations on the use of e-cigarette in smoking cessation, including pregnant and breast-feeding women. This study focused on the use of e-cigarette for smoking cessation. At the request of the SFT, we have used the *fast-track* Delphi process to guide it in the process of reaching a consensus on the place of vaping in the clinical care of people who smoke. Indeed, the SFT is the reference body in the field of smoking cessation for health authorities in France, where the vast majority of its members practice. As a learned society, it promotes the production and sharing of knowledge in this field. In France, the SFT participates with other expert bodies in writing recommendations for the *Haute Autorité de Santé* (HAS), and can also officially propose guidelines that are in line with those of the HAS, or justify any divergences. Its members are not only physicians, but also public health professionals, pharmacists, nurses and midwives [see Additional files 3]. The SFT has no direct links with health decision-makers or government officials. Based on the present results, the SFT will therefore be able to position itself and formulate recommendations.

This *fast-track* Delphi method could also be used to find consensus on their regulation [11, 12, 15, 36]: products contents, price and taxation [37], packaging and labelling, advertising and promotion [38], protection from second-hand exposure [39], etc. The regulation may be challenging as the balance between protecting people who do not smoke, particularly young people, and helping people to quit smoking has proved controversial.

## Conclusions

Conclusions of this *fast-track* Delphi process are useful to guide healthcare professionals to support people quit smoking. Our study shows that the participating French-speaking experts agree that the e-cigarette can help for smoking cessation and harm reduction. It gives some conditions for its use in absence of robust scientific evidence, which can be used as a base to develop clinical guidelines and trainings.

## Supplementary Information

Additional file 1. Step 2 e-questionnaire and step 3 e-questionnaire.

Additional file 2. Typical example of an individualized result for two statements. Expression of the level of agreement with the statement on a 9-point Likert-scale. Agreement: if median (Med.) 7 or more (green tick). Consensus: if interquartile range (IQR) is 3 scale points or less. *n *(%): number (proportion) of respondents to the statement. Mini-plots: distribution of number of responses (top) and summary boxplot (bottom). The expert's personal response is displayed in red in numerical format and using a cursor graphic (middle).

Additional file 3. Partial comparison table between participants (*n*=87) in the *fast-track* Delphi process, versus all SFT members registered in 2023 (*n*=214). Data were not available for the subgroup of members invited to answer step 2 e-questionnaire only (*n*=163; i.e. members who had paid the annual membership as of 5 October 2023).

## Data Availability

The datasets used and/or analysed during the current study are available from the corresponding author on reasonable request.

## Abbreviations

COPD: Chronic Obstructive Pulmonary Disease
DIU: French Inter-University Diploma
HAS: *Haute Autorité de Santé*
IQR: Interquartile Range
NGT: Nominal Group Technique
SFT: French-speaking Society for Smoking Cessation (*Société francophone de tabacologie)*
