# Supplementary material for: Electronic cigarette for smoking cessation: a fast-track Delphi consensus of French-speaking experts
Source: Arch Public Health. 2025 Oct 23;83:260. doi: 10.1186/s13690-025-01725-x (PMC12548246; doi:10.1186/s13690-025-01725-x)
Supplement: Supplementary file 1 — Additional file 1. Step 2 e-questionnaire and step 3 e-questionnaire. [file 13690_2025_1725_MOESM1_ESM.pdf]

unisanté

Centre universitaire de médecine générale et santé publique · Lausanne

Step 2 e-questionnaire

The Delphi process is a method developed by Unisanté for rapidly identifying and quantifying possible consensus between experts on a particular topic. A process was launched with the SFT, who wanted to take part in a fast-track Delphi process to answer the question "What is the place and usefulness of vaping in the clinical management of people who smoke? To do this, an initial session was held with the SFT's Board of Directors and Executive Committee to identify and prioritize the proposed thematic statements to be evaluated. As an SFT member, you have the opportunity to take part in the 2nd and 3rd stages of this process: here is the first of two online questionnaires sent to all SFT members.

You may already have taken part in the first step of this *fast-track* Delphi process, in the form of a brainstorming session according to an adapted version of the nominal group technique on 27 September, and we thank you for that.

You are about to fill in the stage 2 questionnaire containing the thematic statements drawn up with the SFT's Board of Directors and Executive Committee. This questionnaire is the 2nd stage of this fast-track Delphi process. It contains 34 statements, organized into 4 sections (scientific knowledge, advice on clinical management, types of population and miscellaneous). It should take you 30-40 minutes to complete. At the end of each statement, you can leave your comments in free text, in cases where you wish to qualify or justify your choice of answer. You can also suggest additional statements or ask questions at the end of each section. Your answers will be treated with strict confidentiality.

If needed, you can always go back to the previous page. Please leave your comments at the end of each statement, in case you want to qualify or justify your answer. You can also suggest additional statements or ask questions at the end of each section. Your answers will be treated with strict confidentiality.

This questionnaire is based on the following definitions:

> **Electronic cigarette**: electronic nicotine delivery system, also known as e-cigarette, e-cig or vape. Only liquids containing nicotine are considered in this questionnaire. Heated tobacco is a different product that is not considered here (in any of the questions).
>
> **Vaping**: use of the e-cigarette.

In case of technical problems or any other question related to the project, please do not hesitate to contact us at xxx.xxx@xxx.xx.

We thank you in advance!

Please select your gender:

☐Male
☐Female
☐Other

Please select your age group:

☐20 to 29 years old
☐30 to 39 years old
☐40 to 49 years old
☐50 to 59 years old
☐60 years old or more
☐I do not wish to answer

Please select the field that best describes your profession:

☐Physician
☐Nurse
☐Midwife or male midwife
☐Pharmacist
☐Other health or care professional activity

Please select the field that best describes your field of activity:

☐Care
☐Prevention
☐Teaching
☐Research
☐Other

Please select the field that best describes the place of your clinical activity:

☐Hospital center
☐Private practice or general practitioner
☐Health or care center
☐Other

Please select the field that best describes the type of your consultation:

☐Ambulatory consultation
☐In-patients

What tobacco training do you have?

☐Inter-University Diploma (DIU)
☐Other

If necessary, you can add a comment about your training here: (free text)

Please enter your e-mail address: (text)

Please indicate here any conflicts of interest you may have in relation to the theme "vaping in the clinical management of people who smoke: (free text)

Scientific knowledge

Statement 1: The action of the e-cigarette on smoking cessation is well known.

Please indicate your level of agreement with the statement, on a scale from 1 (= total disagreement) to 9 (= total agreement).

☐Total disagreement (1)
☐(2)
☐Disagreement (3)
☐(4)
☐Neutral (5)
☐(6)
☐Agreement (7)
☐(8)
☐Total agreement (9)
☐I do not want or do not wish to state my level of agreement with this statement.

Statement 1 - Comment to qualify or justify your answer [optional]: (free text)

Statement 2: The effectiveness of the e-cigarette in smoking cessation (absticence of 6 months or more) has been scientifically demonstrated.

Please indicate your level of agreement with the statement, on a scale from 1 (= total disagreement) to 9 (= total agreement).

☐Total disagreement (1)
☐(2)
☐Disagreement (3)
☐(4)
☐Neutral (5)

☐(6)
☐Agreement (7)
☐(8)
☐Total agreement (9)
☐I do not want or do not wish to state my level of agreement with this statement.

Statement 2 - Comment to qualify or justify your answer [optional]: (free text)

Statement 3: The benefit-risk ratio of the e-cigarette use in the context of smoking cessation aid is favorable.

Please indicate your level of agreement with the statement, on a scale from 1 (= total disagreement) to 9 (= total agreement).

☐Total disagreement (1)
☐(2)
☐Disagreement (3)
☐(4)
☐Neutral (5)
☐(6)
☐Agreement (7)
☐(8)
☐Total agreement (9)
☐I do not want or do not wish to state my level of agreement with this statement.

Statement 3 - Comment to qualify or justify your answer [optional]: (free text)

Statement 4: The e-cigarette use over several years produces few adverse effects.

Please indicate your level of agreement with the statement, on a scale from 1 (= total disagreement) to 9 (= total agreement).

☐Total disagreement (1)
☐(2)
☐Disagreement (3)
☐(4)
☐Neutral (5)
☐(6)
☐Agreement (7)
☐(8)
☐Total agreement (9)
☐I do not want or do not wish to state my level of agreement with this statement.

Statement 4 - Comment to qualify or justify your answer [optional]: (free text)

Statement 5: nicotine dependence is generally higher in people who smoke than in those who only use electronic cigarette.

Please indicate your level of agreement with the statement, on a scale from 1 (= total disagreement) to 9 (= total agreement).

☐Total disagreement (1)
☐(2)
☐Disagreement (3)
☐(4)
☐Neutral (5)
☐(6)
☐Agreement (7)
☐(8)
☐Total agreement (9)
☐I do not want or do not wish to state my level of agreement with this statement.

Statement 5 - Comment to qualify or justify your answer [optional]: (free text)

Statement 6: The e-cigarette can significantly reduce the risks (morbidity and mortality) of smoking, provided you stop smoking altogether.

Please indicate your level of agreement with the statement, on a scale from 1 (= total disagreement) to 9 (= total agreement).

☐Total disagreement (1)
☐(2)
☐Disagreement (3)
☐(4)
☐Neutral (5)
☐(6)
☐Agreement (7)
☐(8)
☐Total agreement (9)
☐I do not want or do not wish to state my level of agreement with this statement.

Statement 6 - Comment to qualify or justify your answer [optional]: (free text)

Scientific knowledge- Please indicate here, if you want to, any additional comment related to this section [optional]: (free text)

Advice on clinical care

Statement 7: The e-cigarette is an important tool in the clinical care of people who smoke.

Please indicate your level of agreement with the statement, on a scale from 1 (= total disagreement) to 9 (= total agreement).

☐Total disagreement (1)
☐(2)
☐Disagreement (3)
☐(4)
☐Neutral (5)
☐(6)
☐Agreement (7)
☐(8)
☐Total agreement (9)
☐I do not want or do not wish to state my level of agreement with this statement.

Statement 7 - Comment to qualify or justify your answer [optional]: (free text)

Statement 8: The e-cigarette can be used as nicotine replacement therapy.

Please indicate your level of agreement with the statement, on a scale from 1 (= total disagreement) to 9 (= total agreement).

☐Total disagreement (1)
☐(2)
☐Disagreement (3)
☐(4)
☐Neutral (5)
☐(6)
☐Agreement (7)
☐(8)
☐Total agreement (9)
☐I do not want or do not wish to state my level of agreement with this statement.

Statement 8 - Comment to qualify or justify your answer [optional]: (free text)

Statement 9: The e-cigarette has a place and a usefulness in smoking cessation, provided it is used under the right conditions (nicotine dose, type of liquid, electrical resistance, etc.).

Please indicate your level of agreement with the statement, on a scale from 1 (= total disagreement) to 9 (= total agreement).

☐Total disagreement (1)
☐(2)
☐Disagreement (3)
☐(4)
☐Neutral (5)
☐(6)
☐Agreement (7)
☐(8)
☐Total agreement (9)
☐I do not want or do not wish to state my level of agreement with this statement.

Statement 9 - Comment to qualify or justify your answer [optional]: (free text)

Statement 10: The electronic cigarette is a second-line smoking cessation aid, i.e. after pharmacological treatments have failed or proved difficult to use.

Please indicate your level of agreement with the statement, on a scale from 1 (= total disagreement) to 9 (= total agreement).

☐Total disagreement (1)
☐(2)
☐Disagreement (3)
☐(4)
☐Neutral (5)
☐(6)
☐Agreement (7)
☐(8)
☐Total agreement (9)
☐I do not want or do not wish to state my level of agreement with this statement.

Statement 10 - Comment to qualify or justify your answer [optional]: (free text)

Statement 11: The electronic cigarette is a first-line smoking cessation aid when pharmacological treatments are refused.

Please indicate your level of agreement with the statement, on a scale from 1 (= total disagreement) to 9 (= total agreement).

☐Total disagreement (1)
☐(2)
☐Disagreement (3)
☐(4)
☐Neutral (5)
☐(6)
☐Agreement (7)
☐(8)
☐Total agreement (9)
☐I do not want or do not wish to state my level of agreement with this statement.

Statement 11 - Comment to qualify or justify your answer [optional]: (free text)

Statement 12: The electronic cigarette is a first-line smoking cessation aid if you want to vape.

Please indicate your level of agreement with the statement, on a scale from 1 (= total disagreement) to 9 (= total agreement).

☐Total disagreement (1)
☐(2)
☐Disagreement (3)
☐(4)
☐Neutral (5)
☐(6)
☐Agreement (7)
☐(8)
☐Total agreement (9)
☐I do not want or do not wish to state my level of agreement with this statement.

Statement 12 - Comment to qualify or justify your answer [optional]: (free text)

Statement 13: The e-cigarette can be used in association with nicotine replacement products.

Please indicate your level of agreement with the statement, on a scale from 1 (= total disagreement) to 9 (= total agreement).

☐Total disagreement (1)
☐(2)
☐Disagreement (3)
☐(4)
☐Neutral (5)
☐(6)
☐Agreement (7)
☐(8)
☐Total agreement (9)
☐I do not want or do not wish to state my level of agreement with this statement.

Statement 13 - Comment to qualify or justify your answer [optional]: (free text)

Statement 14: The e-cigarette can be recommended for situations where there is a high risk of relapse (e.g. during an aperitif).

Please indicate your level of agreement with the statement, on a scale from 1 (= total disagreement) to 9 (= total agreement).

☐Total disagreement (1)
☐(2)
☐Disagreement (3)
☐(4)
☐Neutral (5)
☐(6)
☐Agreement (7)
☐(8)
☐Total agreement (9)
☐I do not want or do not wish to state my level of agreement with this statement.

Statement 14 - Comment to qualify or justify your answer [optional]: (free text)

Statement 15: The e-cigarette should be used for a limited period, with the aim of quitting in a second phase, once smoking cessation has been consolidated.

Please indicate your level of agreement with the statement, on a scale from 1 (= total disagreement) to 9 (= total agreement).

☐Total disagreement (1)
☐(2)
☐Disagreement (3)
☐(4)
☐Neutral (5)

☐(6)
☐Agreement (7)
☐(8)
☐Total agreement (9)
☐I do not want or do not wish to state my level of agreement with this statement.

Statement 15 - Comment to qualify or justify your answer [optional]: (free text)

Statement 16: The simultaneous use of conventional cigarette and the e-cigarette (dual use) should only be a transitional phase, with the aim of stopping smoking altogether.

Please indicate your level of agreement with the statement, on a scale from 1 (= total disagreement) to 9 (= total agreement).

☐Total disagreement (1)
☐(2)
☐Disagreement (3)
☐(4)
☐Neutral (5)
☐(6)
☐Agreement (7)
☐(8)
☐Total agreement (9)
☐I do not want or do not wish to state my level of agreement with this statement.

Statement 16 - Comment to qualify or justify your answer [optional]: (free text)

Statement 17: Smoking cessation specialists must be trained to provide information as well as medical and technical advice on the use of the e-cigarette.

Please indicate your level of agreement with the statement, on a scale from 1 (= total disagreement) to 9 (= total agreement).

☐Total disagreement (1)
☐(2)
☐Disagreement (3)
☐(4)
☐Neutral (5)
☐(6)
☐Agreement (7)
☐(8)
☐Total agreement (9)
☐I do not want or do not wish to state my level of agreement with this statement.

Statement 17 - Comment to qualify or justify your answer [optional]: (free text)

Statement 18: It is important to recommend increasing the dose of nicotine rather than the power of the e-cigarette.

Please indicate your level of agreement with the statement, on a scale from 1 (= total disagreement)

☐Total disagreement (1)
☐(2)
☐Disagreement (3)
☐(4)
☐Neutral (5)
☐(6)
☐Agreement (7)
☐(8)
☐Total agreement (9)
☐I do not want or do not wish to state my level of agreement with this statement.

Statement 18 - Comment to qualify or justify your answer [optional]: (free text)

Statement 19: Patients should be encouraged to try different flavors.

Please indicate your level of agreement with the statement, on a scale from 1 (= total disagreement) to 9 (= total agreement).

☐Total disagreement (1)
☐(2)
☐Disagreement (3)
☐(4)
☐Neutral (5)
☐(6)
☐Agreement (7)
☐(8)
☐Total agreement (9)
☐I do not want or do not wish to state my level of agreement with this statement.

Statement 19 - Comment to qualify or justify your answer [optional]: (free text)

Statement 20: It is necessary to monitor the undesirable effects of the e-cigarette.

Please indicate your level of agreement with the statement, on a scale from 1 (= total disagreement) to 9 (= total agreement).

☐Total disagreement (1)
☐(2)
☐Disagreement (3)
☐(4)
☐Neutral (5)
☐(6)
☐Agreement (7)
☐(8)
☐Total agreement (9)
☐I do not want or do not wish to state my level of agreement with this statement.

Statement 20 - Comment to qualify or justify your answer [optional]: (free text)

Advice on clinical care- Please indicate here, if you want to, any additional comment related to this section [optional]: (free text)

Population types

Statement 21: The e-cigarette can be recommended for smoking cessation in patients with psychiatric disorders.

Please indicate your level of agreement with the statement, on a scale from 1 (= total disagreement) to 9 (= total agreement).

☐Total disagreement (1)
☐(2)
☐Disagreement (3)
☐(4)
☐Neutral (5)
☐(6)
☐Agreement (7)
☐(8)
☐Total agreement (9)
☐I do not want or do not wish to state my level of agreement with this statement.

Statement 21 - Comment to qualify or justify your answer [optional]: (free text)

Statement 22: The e-cigarette can be recommended for smoking cessation in patients with co-addictions.

Please indicate your level of agreement with the statement, on a scale from 1 (= total disagreement) to 9 (= total agreement).

☐Total disagreement (1)
☐(2)
☐Disagreement (3)
☐(4)
☐Neutral (5)
☐(6)
☐Agreement (7)
☐(8)
☐Total agreement (9)
☐I do not want or do not wish to state my level of agreement with this statement.

Statement 22 - Comment to qualify or justify your answer [optional]: (free text)

Statement 23: The e-cigarette can be recommended for smoking cessation pregnant women.

Please indicate your level of agreement with the statement, on a scale from 1 (= total disagreement) to 9 (= total agreement).

☐Total disagreement (1)
☐(2)
☐Disagreement (3)
☐(4)
☐Neutral (5)
☐(6)
☐Agreement (7)
☐(8)
☐Total agreement (9)
☐I do not want or do not wish to state my level of agreement with this statement.

Statement 23 - Comment to qualify or justify your answer [optional]: (free text)

Statement 24: The e-cigarette can be recommended for smoking cessation in breast-feeding women.

Please indicate your level of agreement with the statement, on a scale from 1 (= total disagreement) to 9 (= total agreement).

☐Total disagreement (1)
☐(2)
☐Disagreement (3)
☐(4)
☐Neutral (5)
☐(6)
☐Agreement (7)
☐(8)
☐Total agreement (9)
☐I do not want or do not wish to state my level of agreement with this statement.

Statement 24 - Comment to qualify or justify your answer [optional]: (free text)

Statement 25: A pregnant woman who has quitted smoking with an e-cigarette should not be discouraged from using it if there is a risk of relapse.

Please indicate your level of agreement with the statement, on a scale from 1 (= total disagreement) to 9 (= total agreement).

☐Total disagreement (1)
☐(2)
☐Disagreement (3)
☐(4)
☐Neutral (5)
☐(6)
☐Agreement (7)
☐(8)
☐Total agreement (9)
☐I do not want or do not wish to state my level of agreement with this statement.

Statement 25 - Comment to qualify or justify your answer [optional]: (free text)

Statement 26: A breast-feeding woman who has quitted smoking with an e-cigarette should not be discouraged from using it if there is a risk of relapse.

Please indicate your level of agreement with the statement, on a scale from 1 (= total disagreement) to 9 (= total agreement).

☐Total disagreement (1)
☐(2)
☐Disagreement (3)
☐(4)
☐Neutral (5)

☐(6)
☐Agreement (7)
☐(8)
☐Total agreement (9)
☐I do not want or do not wish to state my level of agreement with this statement.

Statement 26 - Comment to qualify or justify your answer [optional]: (free text)

Statement 27: The e-cigarette can be recommended for peri-operative smoking cessation (pre-and post-operative).

Please indicate your level of agreement with the statement, on a scale from 1 (= total disagreement) to 9 (= total agreement).

☐Total disagreement (1)
☐(2)
☐Disagreement (3)
☐(4)
☐Neutral (5)
☐(6)
☐Agreement (7)
☐(8)
☐Total agreement (9)
☐I do not want or do not wish to state my level of agreement with this statement.

Statement 27 - Comment to qualify or justify your answer [optional]: (free text)

Statement 28: The e-cigarette can be recommended for smoking cessation in hospitalized patients.

Please indicate your level of agreement with the statement, on a scale from 1 (= total disagreement) to 9 (= total agreement).

☐Total disagreement (1)
☐(2)
☐Disagreement (3)
☐(4)
☐Neutral (5)
☐(6)
☐Agreement (7)
☐(8)
☐Total agreement (9)
☐I do not want or do not wish to state my level of agreement with this statement.

Statement 28 - Comment to qualify or justify your answer [optional]: (free text)

Statement 29: The e-cigarette can be recommended for smoking cessation in coronary patients.

Please indicate your level of agreement with the statement, on a scale from 1 (= total disagreement) to 9 (= total agreement).

☐Total disagreement (1)
☐(2)
☐Disagreement (3)
☐(4)
☐Neutral (5)
☐(6)
☐Agreement (7)
☐(8)
☐Total agreement (9)
☐I do not want or do not wish to state my level of agreement with this statement.

Statement 29 - Comment to qualify or justify your answer [optional]: (free text)

Statement 30: The e-cigarette can be recommended for smoking cessation in COPD patients.

Please indicate your level of agreement with the statement, on a scale from 1 (= total disagreement) to 9 (= total agreement).

☐Total disagreement (1)
☐(2)
☐Disagreement (3)
☐(4)
☐Neutral (5)
☐(6)
☐Agreement (7)
☐(8)
☐Total agreement (9)
☐I do not want or do not wish to state my level of agreement with this statement.

Statement 30 - Comment to qualify or justify your answer [optional]: (free text)

Statement 31: The e-cigarette can be recommended for smoking cessation in patients under 18.

Please indicate your level of agreement with the statement, on a scale from 1 (= total disagreement) to 9 (= total agreement).

☐Total disagreement (1)
☐(2)
☐Disagreement (3)
☐(4)
☐Neutral (5)
☐(6)
☐Agreement (7)
☐(8)
☐Total agreement (9)
☐I do not want or do not wish to state my level of agreement with this statement.

Statement 31 - Comment to qualify or justify your answer [optional]: (free text)

Population type - Please indicate here, if you want to, any additional comment related to this section [optional]: (free text)

Miscellaneous

Statement 32: The e-cigarette should only be sold in pharmacies.

Please indicate your level of agreement with the statement, on a scale from 1 (= total disagreement) to 9 (= total agreement).

☐Total disagreement (1)
☐(2)
☐Disagreement (3)
☐(4)
☐Neutral (5)
☐(6)
☐Agreement (7)
☐(8)
☐Total agreement (9)
☐I do not want or do not wish to state my level of agreement with this statement.

Statement 32 - Comment to qualify or justify your answer [optional]: (free text)

Statement 33: There is a need for clinical guidelines on the use of the e-cigarette for smoking cessation.

Please indicate your level of agreement with the statement, on a scale from 1 (= total disagreement) to 9 (= total agreement).

☐Total disagreement (1)
☐(2)
☐Disagreement (3)
☐(4)
☐Neutral (5)
☐(6)
☐Agreement (7)
☐(8)
☐Total agreement (9)
☐I do not want or do not wish to state my level of agreement with this statement.

Statement 33 - Comment to qualify or justify your answer [optional]: (free text)

Statement 34: Clinical recommendations should distinguish between products from the tobacco industry and those from independent manufacturers.

Please indicate your level of agreement with the statement, on a scale from 1 (= total disagreement) to 9 (= total agreement).

☐Total disagreement (1)
☐(2)
☐Disagreement (3)
☐(4)
☐Neutral (5)
☐(6)
☐Agreement (7)
☐(8)
☐Total agreement (9)
☐I do not want or do not wish to state my level of agreement with this statement.

Statement 34 - Comment to qualify or justify your answer [optional]: (free text)

Miscellaneous - Please indicate here, if you want to, any additional comment related to this section [optional]: (free text)

Step 3 e-questionnaire

You have taken part in the second step of this *fast-track Delphi* process a few days ago. We would like to thank you very much for your participation so far. It identified 74% of statements that had already reached agreement and consensus.

This questionnaire is the third and final step of this *fast-track Delphi* process. It contains only statements that did not meet the required level of agreement and/or consensus, or for which comments suggested the need to reformulate, combine or separate them. It contains 8 statements divided into 2 sections. It should take you between 10-20 minutes to complete.

We ask you to complete this questionnaire in relation to the results of the previous step, which were sent to you by email (Word document). It is not mandatory that you change your mind; it is simply a matter of questioning your opinion in light of the group results generated in the previous step.

This questionnaire is based on the following definitions:

> **Electronic cigarette**: electronic nicotine delivery system, also known as e-cigarette, e-cig or vape. Only liquids containing nicotine are considered in this questionnaire. Heated tobacco is a different product that is not considered here (in any of the questions).
>
> **Vaping**: use of the e-cigarette.

Please leave your comments at the end of each statement in case you wish to qualify or justify your choice of answer. You can also write a general comment at the end of each section. Your answers will be treated with strict confidentiality criteria. If necessary, you can always go back to the previous page.

In case of technical problems or any other question related to the project, do not hesitate to contact us at xxx.xxx@xxx.xx.

We thank you in advance!

Scientific knowledge

Statement 1: According to current data, the e-cigarette is effective for smoking cessation (abstinence of 6 months or more).

This statement has been modified from your responses and comments, based on statements 2 from step 2 e-questionnaire (see pages 5 and 7-8 of your individualized result report).

Please indicate your level of agreement with the statement, on a scale from 1 (= total disagreement) to 9 (= total agreement).

☐Total disagreement (1)
☐(2)

☐Disagreement (3)
☐(4)
☐Neutral (5)
☐(6)
☐Agreement (7)
☐(8)
☐Total agreement (9)
☐I do not want or do not wish to state my level of agreement with this statement.

Statement 1 - Comment to qualify or justify your answer [optional]: (free text)

Statement 2: According to current data, the e-cigarette use over several years produces few severe adverse effects.

This statement has been modified from your responses and comments, based on statements 2 from step 2 e-questionnaire (see pages 5 and 9-11 of your individualized result report).

Please indicate your level of agreement with the statement, on a scale from 1 (= total disagreement) to 9 (= total agreement).

☐Total disagreement (1)
☐(2)
☐Disagreement (3)
☐(4)
☐Neutral (5)
☐(6)
☐Agreement (7)
☐(8)
☐Total agreement (9)
☐I do not want or do not wish to state my level of agreement with this statement.

Statement 2 - Comment to qualify or justify your answer [optional]: (free text)

Statement 3: Nicotine dependence is comparable between people who smoke and recent e-cigarette users.

This statement has been modified from your responses and comments, based on statements 2 from step 2 e-questionnaire (see pages 5 and 11-12 of your individualized result report).

Please indicate your level of agreement with the statement, on a scale from 1 (= total disagreement) to 9 (= total agreement).

☐Total disagreement (1)
☐(2)

☐Disagreement (3)
☐(4)
☐Neutral (5)
☐(6)
☐Agreement (7)
☐(8)
☐Total agreement (9)
☐I do not want or do not wish to state my level of agreement with this statement.

Statement 3 - Comment to qualify or justify your answer [optional]: (free text)

Statement 4: The e-cigarette is very likely to reduce the risks (morbidity and mortality) of smoking, provided you stop smoking altogether.

This statement has been modified from your responses and comments, based on statements 2 from step 2 e-questionnaire (see pages 5 and 12-13 of your individualized result report).

Please indicate your level of agreement with the statement, on a scale from 1 (= total disagreement) to 9 (= total agreement).

☐Total disagreement (1)
☐(2)
☐Disagreement (3)
☐(4)
☐Neutral (5)
☐(6)
☐Agreement (7)
☐(8)
☐Total agreement (9)
☐I do not want or do not wish to state my level of agreement with this statement.

Statement 4 - Comment to qualify or justify your answer [optional]: (free text)

Scientific knowledge - Please indicate here, if you want to, any additional comment related to this section [optional]: (free text)

Advice on clinical care

Statement 5: Pharmacological treatments and the e-cigarette need to be presented to people who smoke, with clear explanations of their advantages and disadvantages, and then the person who smokes needs to be supported in their choice (shared decision making).

This statement has been modified from your responses and comments, based on statements 2 from step 2 e-questionnaire (see pages 16 and 22-24 of your individualized result report).

Please indicate your level of agreement with the statement, on a scale from 1 (= total disagreement) to 9 (= total agreement).

☐Total disagreement (1)
☐(2)
☐Disagreement (3)
☐(4)
☐Neutral (5)
☐(6)
☐Agreement (7)
☐(8)
☐Total agreement (9)
☐I do not want or do not wish to state my level of agreement with this statement.

Statement 5 - Comment to qualify or justify your answer [optional]: (free text)

Statement 6: The e-cigarette is an option for helping people to stop smoking, when they want to vape or refuse pharmacological treatments.

This statement has been modified from your responses and comments, based on statements 11 and 12 from step 2 e-questionnaire (see pages 16 and 24-26 of your individualized result report).

Please indicate your level of agreement with the statement, on a scale from 1 (= total disagreement) to 9 (= total agreement).

☐Total disagreement (1)
☐(2)
☐Disagreement (3)
☐(4)
☐Neutral (5)
☐(6)
☐Agreement (7)
☐(8)
☐Total agreement (9)

☐I do not want or do not wish to state my level of agreement with this statement.

Statement 6 - Comment to qualify or justify your answer [optional]: (free text)

Statement 7: The e-cigarette can be recommended in situations where there is a risk of relapse, such as when going out in the presence of other people who smoke.

This statement has been modified from your responses and comments, based on statements 2 from step 2 e-questionnaire (see pages 17 and 27-28 of your individualized result report).

Please indicate your level of agreement with the statement, on a scale from 1 (= total disagreement) to 9 (= total agreement).

☐Total disagreement (1)
☐(2)
☐Disagreement (3)
☐(4)
☐Neutral (5)
☐(6)
☐Agreement (7)
☐(8)
☐Total agreement (9)
☐I do not want or do not wish to state my level of agreement with this statement.

Statement 7 - Comment to qualify or justify your answer [optional]: (free text)

Statement 8: In the event of withdrawal symptoms when using an e-cigarette, it is recommended to increase the nicotine concentration, not its electrical power.

This statement has been modified from your responses and comments, based on statements 18 from step 2 e-questionnaire (see pages 17 and 31-32 of your individualized result report).

Please indicate your level of agreement with the statement, on a scale from 1 (= total disagreement) to 9 (= total agreement).

☐Total disagreement (1)
☐(2)
☐Disagreement (3)
☐(4)
☐Neutral (5)
☐(6)
☐Agreement (7)
☐(8)
☐Total agreement (9)

☐I do not want or do not wish to state my level of agreement with this statement.

Statement 8 - Comment to qualify or justify your answer [optional]: (free text)

Advice on clinical care - Please indicate here, if you want to, any additional comment related to this section [optional]: (free text)
