# Supplementary figures and images for: Electronic cigarette for smoking cessation: a fast-track Delphi consensus of French-speaking experts
Source: Arch Public Health. 2025 Oct 23;83:260. doi: 10.1186/s13690-025-01725-x (PMC12548246; doi:10.1186/s13690-025-01725-x)

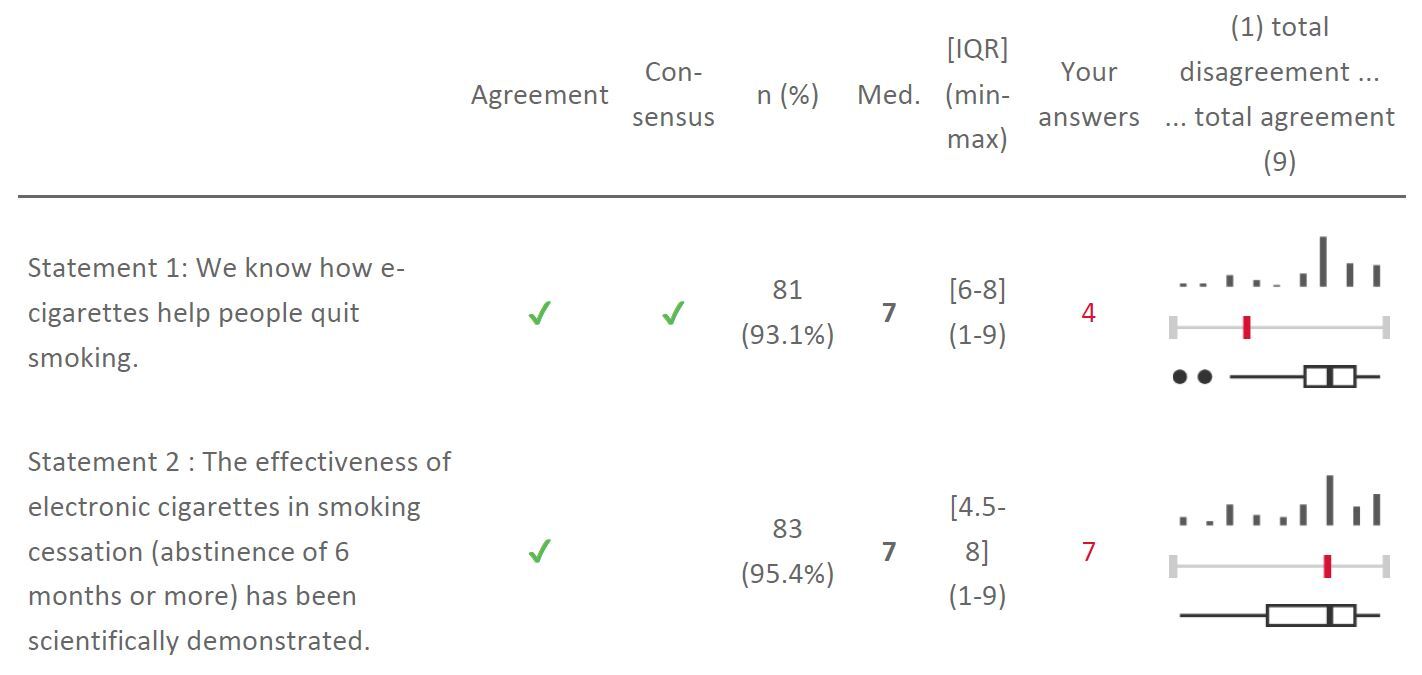

Supplement: Supplementary file 2 — Additional file 2. Typical example of an individualized result for two statements. Expression of the level of agreement with the statement on a 9-point Likert-scale. Agreement: if median (Med.) 7 or more (green tick). Consensus: if interquartile range (IQR) is 3 scale points or less. n (%): number (proportion) of respondents to the statement. Mini-plots: distribution of number of responses (top) and summary boxplot (bottom). The expert's personal response is displayed in red in numerical format and using a cursor graphic (middle). [file 13690_2025_1725_MOESM2_ESM.jpg]
