# Supplementary material for: Electronic cigarette for smoking cessation: a fast-track Delphi consensus of French-speaking experts
Source: Arch Public Health. 2025 Oct 23;83:260. doi: 10.1186/s13690-025-01725-x (PMC12548246; doi:10.1186/s13690-025-01725-x)
Supplement: Supplementary file 3 — Additional file 3. Partial comparison table between participants (n=87) in the fast-track Delphi process, versus all SFT members registered in 2023 (n=214). Data were not available for the subgroup of members invited to answer step 2 e-questionnaire only (n=163; i.e. members who had paid the annual membership as of 5 October 2023). [file 13690_2025_1725_MOESM3_ESM.docx]

|  | Respondents of the questionnaires | | Total SFT members | |
| --- | --- | --- | --- | --- |
|  | n  (N=87) | Proportion | n  (N=214) | Proportion |
| Gender |  |  |  |  |
| Female | 53 | 60.9% | 147 | 68.7% |
| Male | 34 | 39.1% | 65 | 30.4% |
| Age |  |  |  |  |
| ≥ 60 years old | 37 | 42.5% | 83 | 38.8% |
| 50 to 59 years old | 22 | 25.3% | 51 | 23.8% |
| 40 to 49 years old | 17 | 19.5% | 50 | 23.4% |
| 30 to 39 years old | 9 | 10.3% | 29 | 13.6% |
| 20 to 29 years old | 1 | 1.1% | 1 | 0.5% |
| I do not wish to answer^a^ | 1 | 1.1% | N.A. | N.A. |
| Professional activity |  |  |  |  |
| Physician | 61 | 70.1% | 145 | 67.8% |
| Other health or care professional activity | 10 | 11.5% | 12 | 5.6% |
| Nurse | 7 | 8.0% | 24 | 11.2% |
| Midwife or male midwife | 7 | 8.0% | 18 | 8.4% |
| Pharmacist | 2 | 2.3% | 6 | 2.8% |
| Activities *^, a^ |  |  |  |  |
| Care | 74 | 85.1% | N.A. | N.A. |
| Prevention | 36 | 41.4% | N.A. | N.A. |
| Teaching | 32 | 36.8% | N.A. | N.A. |
| Research | 17 | 19.5% | N.A. | N.A. |
| Other | 5 | 5.7% | N.A. | N.A. |
| Place of clinical activity * |  |  |  |  |
| Hospital center | 56 | 64.4% | 115 | 53.7% |
| Private practice or general practitioner | 20 | 23.0% | 38 | 17.8% |
| Health or care center | 10 | 11.5% | 5 | 2.3% |
| Other | 8 | 9.2% | 56 | 26.2% |
| Type of consultation *^, a^ |  |  |  |  |
| Ambulatory consultation | 54 | 62.1% | N.A. | N.A. |
| In-patients | 37 | 42.5% | N.A. | N.A. |
| Training in smoking cessation *^, a^ |  |  |  |  |
| Inter-University Diploma (DIU) | 67 | 77.0% | N.A. | N.A. |
| Other | 28 | 32.2% | N.A. | N.A. |
| Country of main professional activity |  |  |  |  |
| France | 80 | 92.0% | 195 | 91.1% |
| Belgium | 5 | 5.7% | 10 | 4.7% |
| Switzerland | 1 | 1.1% | 3 | 1.4% |
| Other | 1 | 1.1% | 6 | 2.8% |

Proportions are calculated on the total of respondents.

SFT: French-speaking Society for Smoking Cessation (*Société francophone de tabacologie*).

* Several answers possible - the sum of the proportions may exceed 100%.

^a^ Data not collected at SFT membership subscription.
